# What is the influence of tonsillectomy on the level of periodontal pathogens on the tongue dorsum and in periodontal pockets

**DOI:** 10.1186/s12903-018-0521-7

**Published:** 2018-04-06

**Authors:** V. N. Diener, A. Gay, M. B. Soyka, T. Attin, P. R. Schmidlin, P. Sahrmann

**Affiliations:** 1 Clinic for Preventive Dentistry, Periodontology and Cariologiy, Center of Dental Medicine, University of Zurich, Zurich, Switzerland; 2 Department of Otolaryngology, Head and Neck Surgery, University Hospital of Zurich, Zurich, Switzerland

**Keywords:** Tonsillectomy, Oral niches, Full mouth disinfection, Periodontal therapy, Bacteria

## Abstract

**Background:**

For periodontal treatment, the full mouth disinfection approach suggests disinfection of oral soft tissues, such as tongue and tonsils concomitant to scaling and root planning since patients might benefit from treatment of these oral niches either. Periodontopathogenes in tonsillar tissue support this hypothesis. This prospective controlled clinical study investigated the change in the oral flora of patients who underwent tonsillectomy. Pockets were tested for eleven bacterial species before and six weeks after the surgical intervention.

**Methods:**

Fifty generally healthy adults were included in this study. The test group consisted of 25 patients with tonsillectomy. The control group included 25 patients with otorhinolarynologic surgery without involvement of the oral cavity. Clinical parameters such as probing pocket depth, bleeding-on-probing index and plaque index were registered the evening before surgery. Also bacterial samples from the gingival sulcus and dorsum linguae were taken, and an additional sample from the removed tonsils in the test group. Six weeks after the intervention microbial samples of pockets and tongue were taken again. Data were tested for significant differences using Wilcoxon rank and Whitney-u-test.

**Results:**

No relevant intra- or intergroup differences were found for the change of the eleven investigated species.

**Conclusion:**

Based on the results of the present study, tonsillectomy does not seem to have an immediate relevant effect on the bacterial flora of tongue or periodontium. This study design was approved by the ethical committee of Zurich (KEK-ZH-Nr.2013–0419).

**Trial registration:**

The trial was retrospectively registered in the German Clinical Trials Register (DRK00014077) on February 20, 2018.

## Background

Periodontitis is an inflammation of hard and soft tissues around the teeth that leads to progressive destruction of the alveolar bone and the fibrous apparatus [1]. Microorganisms located in subgingival biofilm are generally accepted as the main etiologic factor for periodontitis [2]. Especially species of the so-called red complex like *Porphyromonas gingivalis, Tanerella forsythia* and *Treponema denticola* have been shown to be associated with chronic periodontitis [3, 4]. Socransky and co-workers observed that species of the orange complex, among others, like *Prevotella intermedia, Fusobacterium nucleatum, Peptostreptococcus micros, Eubacterium nodatum* and *Campylobacter rectus* can be considered as early colonizers of the periodontium before species of the red complex can be found. Likewise, the presence of *Aggregatibacter actinomycetemcomitans* is associated with periodontal inflammation. The latter species has been suggested to play an important role in more aggressive forms of periodontitis [1, 5]. While bacteria represent the primary etiologic factor of this biofilm-associated disease, progression is aggravated by environmental and host factors such as tobacco abuse [6, 7], general diseases like diabetes mellitus [8, 9] and the individual immune response [10, 11]. Any tissue destruction defined as periodontitis is clinically characterized by the formation of periodontal pockets with measurable probing pocket depths ≥4 mm, marginal inflammation, optional pus secretion and clinical loss of attachment [12]. If periodontitis remains untreated, teeth continue to lose attachment until they have to be extracted or even exfoliate [13]. Data regarding the prevalence of periodontitis vary considerably due to different definitions of periodontitis, a lack of standardized measurement techniques and different ethnic and social backgrounds [14]. However, prevalences between 10%–50% strongly depending on the disease classification applied and the population assessed is indicated in the scientific literature [15–17].

Periodontitis therapy primarily aims at arresting periodontal destruction by controlling the inflammation. Therefore, pathogenic biofilm is removed and the patient is instructed with effective oral hygiene measures in order to reduce or even eliminate any new biofilm formation [11]. At periodontally diseased sites, where clinical probing depth is increased and the root surfaces lost their fibrous attachment to the bone, mechanical debridement is performed. Since it is not possible to reach the complete affected root surface neither by conventional root debridement nor by surgical access, several additive measures have been proposed to improve the therapeutic effect, including the use of pharmaceutics or special instrumentations like laser application [18] or air powder abrasives [19]. Other than systemic antibiotics [1], the application of topical antiseptics i.e. PVP-iodine or chlorhexidine [11, 20] have been assessed. Based on the widespread use of chlorhexidine in dentistry Quirynen et al. [21] introduced an approach called Full Mouth Disinfection (FMD), which aimed to disinfect all the so-called “intraoral niches” beyond periodontal pockets, namely the tonsils and the dorsum of the tongue. According to the FMD philosophy this is important in order to prevent quick microbial reinfection of previously treated root surfaces from these niches where biofilm can persist. Therefore a full-mouth scaling of all subgingival areas within 24 h was proposed, followed by repeated disinfection of the tonsils and the dorsum of the tongue as well as of all affected subgingival pockets [21].

With regard to the re-establishment of biofilms with bacteria of pathogenic potential, the role of the palatine tonsils seems of special importance [22], as they often harbour mineralized tonsilloliths, which may act as non-shedding surfaces [23, 24]. Tonsilloliths are formed in crypts, i.e. deep plications in the chronically inflamed tonsils [25]. On the latter, mature biofilms with a great variety of different species and a considerable proportion of anaerobe bacterial flora have been found [26]. In fact, periodontal key pathogens such as *Aggregatibacter actinomycetemcomitans*, *Porphyromonas* spp., *Prevotella intermedia*, *Campylobacter rectus*, *Eubacterium nodatum*, *Fusobacterium nucleatum*, *Prevotella melaninogenica* and *Peptostreptococcus micros* have already been isolated in tonsillary tissue [26–29].

Despite the fact that FMD has been shown to result in modestly relevant but nevertheless statistically significant clinical benefits compared to quadrantwise subgingival debridement without the use of any antiseptics [30] the actual role of the disinfection of the niches is still unclear [31]. Likewise - and despite the biological plausibility of this theory - the clinical relevance of a possible reinfection from the tonsillar bacterial reservoir on either the oral microbiome or the progression of periodontitis has not been investigated in details so far [32]. Therefore, it was the aim of the present study to assess the effect of tonsillectomy on the taxa of periodontal key microbiota. Our hypothesis was that tonsillectomy would decrease the taxa of the periodontal key bacteria.

## Methods

The study was designed as a controlled prospective clinical investigation. The study design was approved by the liable ethical committee of Zurich (KEK-ZH-Nr.2013–0419, Oct. 17 2013). All study-related examinations were performed in accordance to the declaration of Helsinki as revised in 2000 [33].

### Patient selection

For inclusion, all patients had to be over 18 years with the intellectual and linguistic ability to give their informed consent for study participation. The patients had to be generally healthy with the only exception of an otorhinolaryngologic affliction: Test group patients had to be scheduled for tonsillectomy and control group patients for any ambulant otorhinolarynologic surgery without involvement of the oral cavity.

Exclusion criteria were systemic antibiotic therapy during the preceding three months and the customary use of antiseptic mouth rinses. According to the study protocol, 50 patients were planned to be included, 25 in each the test and the control group.

The responsible otorhinolaryngologist (MBS) contacted suitable patients from the University Hospital of Zurich, Department of Otorhinolaryngology, Head and Neck Surgery, and invited them to participate in the study. Possible participants were informed about the strictly facultative character of the participation. Patients were also informed about aim and nature of the study. All participants had to consider study participation at least 24 h before the intervention and enough time was left for the patients to ask questions before they signed the written patient information and certificate of consent.

All patients received their treatment at the Department of Otolaryngology, Head and Neck Surgery of the University Hospital of Zurich.

### Data acquisition

The baseline appointment took place the evening before the surgical intervention at the hospital. At that time patients gave their written informed consent. They were asked to answer a questionnaire about personal oral hygiene practice, smoking habits, co-morbidities and intake of drugs. In accordance with the otorhinolaryngological team patients were also asked not to change their oral hygiene measures, especially not to use any antiseptic mouthwash after the intervention. Then complete data acquisition and clinical assessment was performed, both by the same investigator (VND) for all patients. The periodontal status was assessed, which included the measurement of clinical periodontal pocket depths (PPD) on six sites of each tooth using a periodontal probe (HH12, Deppeler SA, 1180 Rolle, Switzerland). Additionally, the bleeding-on-probing index (BoP) [34] was recorded at the same sites. Microbiological samples were taken with a paper point (ISO 50, Hain lifesciences GmbH, 72,147 Nehren, Germany) from the deepest periodontal pocket of each quadrant. In the absence of pocket depths exceeding 3 mm, the sample was taken from the mesio-oral site of the first upper molar and one from the mesio-buccal site of the first lower molar. Therefore, the supragingival tooth surface coronal of the deepest pocket was cleaned with a cotton pellet to ensure that only subgingival bacteria would be collected. A sterile paper point was inserted with tweezers to the fundus of the pocket and kept there for 10 s. Care was taken in order to safely prevent saliva contamination using cotton roles on the vestibular and lingual side. In addition, in each patient another microbiological sample was taken from the tongue dorsum by dipping a paper point for 10 s in the middle the proximal tongues’ dorsum. Consequently the paper points were placed into a code-labelled Eppendorf tube and shipped for the laboratory assessment (heico Dent GmbH, 8633 Wolfhausen, Switzerland) the following day. Finally, the teeth were stained with an erythrosine pellet (Esro AG, 8802 Kilchberg, Switzerland) in order to reveal tooth surfaces covered by bacterial plaque. After extensive water rinsing of the mouth the plaque index [35] of all four quadrants was recorded except for the four previously cleaned areas.

The following day during surgery, a small tissue sample was taken from the excised tonsil from the subjects of the test group and stored in physiologic sodium chloride solution in an Eppendorf cup. A sterile paper tip was swabbed over this tissue and was taken for additional bacterial assessment.

### Follow-up appointment

Six weeks after surgery, patients were invited to the Center of Dental Medicine of the University of Zurich. After an update of the specific patient history regarding drug intake and any potential changes in oral hygiene after the intervention, microbiological samples from the same periodontal sites and the tongues’ dorsum were taken and analyzed.

### Laboratory analysis

Bacterial samples were sent to the aforementioned laboratory the day after the examinations. Laboratory analysis was performed blinded with regard to group allocation and appointment number. All bacterial samples were assessed by the microIDent®plus test (Hain lifesciences GmbH, 72,147 Nehren, Germany) using DNA-strip technology, by which eleven key pathogenes were assessed: *Aggregatibacter actinomycetemcomitans*, *Porphyromonas gingivalis*, *Tannerella forsythia*, *Treponema denticola*, *Prevotella intermedia*, *Peptostreptococcus micros*, *Fusobacterium nucleatum*, *Campylobacter rectus*, *Eubacterium nodatum*, *Eikenella corrodens*, *Capnocytophaga* spp. During the laboratory evaluation process, sampled DNA and RNA were isolated and amplified. The semiquantitative analysis for the specific species was performed by comparison with a five-stage colour index. Detailed information about the laboratory process has been described elsewhere [36].

The assessment report depicted a semi-quantitative analysis with a detection threshold of 10^4^ bacteria for all bacteria except *Aggregatibacter actinomycetemcomitans,* for which the threshold was 10^3^. A scheme of the graduation of the five scores is depicted in Table 1.

**Table 1:** Data reporting from the laboratory test and numbers used for statistical calculation

Score assigned by laboratory	According range of bacterial species (for *A. actinomycetemcomitans*)	Estimated counts as used for statistical analysis (for *A. actinomycetemcomitans*)
–	< 10^4^ (<10e^3^)	0.1 (0.1)
(+)	10^4^ (10^3^)	10,000 (1000)
+	< 10^5^ (< 10^4^)	50,000 (5000)
++	< 10^6^ (< 10^5^)	500,000 (50000)
+++	≥10^6^ (≥10^5^)	1,000,000 (500000)

Values for *A. actinomycetemcomitans* is depicted in brackets

### Statistical analysis

In order to enable numerical analysis, the five scores were replaced by estimated counts according to Table 1. All data were analysed by IBM SPSS, Version 22.0, Chicago, IL, USA.

Descriptive analysis of patient characteristics including age, gender, plaque index, bleeding on probing, number of pockets exceeding 4 mm and smoking were calculated.

Data were assessed for normal distribution and in case of a non-parametric dataset as affirmed by both the Shapiro-Wilks and Kolmogorov-Smirnov test. The Mann-Whitney-U test for the assessment of intergroup differences and the Wilcoxon test for intragroup differences was performed.

Dichotomous data for absence/presence of bacterial species or complexes were tested by Pearson’s chi-square test. For all tests, the level of significance was set at 0.05.

## Results

Fifty patients were planned to be included in the study and to be allocated to test (*N* = 25) and control group (N = 25) of this prospective controlled clinical trial. In total, ten patients, four from the test and six from the control group, dropped out from the study since they did not show up for the second appointment. Their data were not included in the following analyses. Patient characteristics at baseline (Table 2) showed statistically significant differences for the patients’ age (*p* = 0.001) and the number of periodontal pockets > 4 mm (*p* = 0.004). For both parameters the control group showed higher values.

**Table 2:** Patient characteristics at baseline

	Test	Control	*p*-value
Age [years]^a^	24.7 ± 7.8	35.3 ± 14.0	**0.001**
Gender [% male]^b^	48.0	64.0	0.393
Plaque index [%]^a^	35.1 ± 10.2	30.6 ± 13.5	0.128
Bleeding on probing [%]^a^	15.8 ± 7.9	20.9 ± 14.0	0.145
Number of PPD >4mm^a^	0.6 ± 2.1	3.4 ± 8.7	**0.004**
Smoker [%]^b^	42	58	0.38

Statistically differences between test and control groups are given in bold characters

^a^Mann-Whitney-U-test

^b^Chi-square-test

*PPD* periodontal pocket depths

Regarding the interventions performed in the control group, septorhinoplasty was performed eleven times, paranasal sinus surgery six times, septoplasty three times, two times turbinoplasty and interventions in the middle ear respectively, and one cochlear implant.

Table 3 shows dichotomously the presence of *A. actinomycetemcomitans*, red complex bacteria and orange complex bacteria in both groups at baseline given in percentages. Cases with a positive detection for *A. actinomycetemcomitans* were significantly more often in the control group while no difference was found for bacterial species of the red or the orange complex between the groups. As tonsil samples had been taken from the excisions in the test group only, no respective data for the control group could be generated.

**Table 3:** Percentile presence of the red complex, orange complex and *A. actinomycetemcomitas* at baseline

Sample origin	Test group			Control group		
	Red complex	Orange complex	*A.a.*	Red complex	Orange complex	*A.a.*
Tonsil	60	64	0	nd	nd	nd
Sulcus	44	100	4	84	100	24
Tongue	52	88	4	80	100	24
Overall presence	84	100	4^A^	88	100	28^A^

^A^ Significant difference between test and control group (*p*-value = 0.049, Chi-square-test)

*Nd* no data, *A.a* Aggregatibacter actinomycetemcomitans

Assessing the semi-quantitative report for the different taxa from the different sites at baseline we found differences for estimated numbers of several species between the groups (Table 4), including *A. actinomycetemcomitans* and the red complex species *T. forsythia* and *T. denticola*. In all of these cases the control patients showed again a higher number of these taxa.

**Table 4:** Significantly different inter-group values for species from different sampling sites at baseline

		*p*-value
Sulcus	*A. actinomycetemcomitans*	0.039
	*T. forsythia*	0.007
	*T. denticola*	0.002
	*P. micros*	0.039
	*C. rectus*	0.020
	*E. nodatum*	0.020
Tongue	*A. actinomycetemcomitans*	0.046
	*T. forsytha*	0.008
	*T. denticola*	0.003
	*P. intermedia*	0.020
	*P. micros*	0.001
	*C. rectus*	0.008
	*E. corrodens*	0.006
	*Capnocytophaga* species	0.024
Total	*A. actinomycetemcomitans*	0.020
	*T. denticola*	0.007

In all listed cases the taxa were higher in the control group (Mann-Whitney-U-test)

From the remaining, 12 out of 40 took antibiotics due to post-interventional complications. This represents 50% of the test group and 29% of the control group. Information about the administered antibiotics is given in Table 5.

**Table 5:** Administered antibiotics in the test group

Agent	Product name	Dose (range)	Number of patients	Indication
Amoxicillin	Augmentin	5–9 days	8 (7 test, 1 control)	Postoperative infection and haemorrhage
Cefuroxim	Zinacef	1.5 g once	1 (control)	Prophylaxis
Clindamycin	Dalacin	8 days	1 (test)	Superinfection
Ceftriaxon	Rocephin	2 g once	1 (control)	Prophylaxis
Ciprofloxacin	Ciproxin	10 days	1 (control)	Postoperative Infection

Assessing the semi-quantitative values for different bacterial species from different sites, several statistically significant differences were found between the groups after 6 weeks: *T. denticola, P. intermedia, C. rectus and E. corrodens,* were reduced significantly more in the sulcus of the test group while *P. intermedia* and *P.micros* was reduced significantly more on the tongue dorsum of the test group (Table 6). However, regarding the pronounced reduction of these species in the test group no association was found between the different sampling sites.

**Table 6:** Significantly different taxa and respective *p*-values for the different sampling sites 6 weeks after intervention

		*p*-value
Sulcus	*T. denticola*	< 0.001
	*P. intermedia*	0.006
	*C. rectus*	0.006
	*E. corredens*	0.023
Tongue	*P. intermedia*	0.017
	*P. micros*	0.029
Total	*T. denticola*	0.003
	*P. intermedia*	0.003
	*P. micros*	0.034
	*F. nucleatum*	0.044
	*C. rectus*	0.011

In addition, we also calculated the differences for the semi-quantitative bacterial taxa between first and second analysis and checked this data for possible differences between test and control group. However, no differences were found. When testing for possible intra-group changes in the taxa from baseline to 6 weeks, we observed significant reductions for *Capnocytophaga* spp. (*p* = 0.003) from the tongue in the control group and for the detection of *T. forsythia* (*p* = 0.36) and *P. gingivalis* (*p* = 0.25) from the tongue in the test group, but no change for species from the sulcus.

Likewise, a reduction below detection limit was significantly more frequent for *A.actinomycetemcomitans* from the tongue and the teeth (*p* = 0.001 and 0.004) in the control group and the red complex (p = < 0.001) and *A.actinomycetemcomitans* (*p* = 0.048) from the teeth for the test group as compared to the respective other group.

**Fig. 1:**
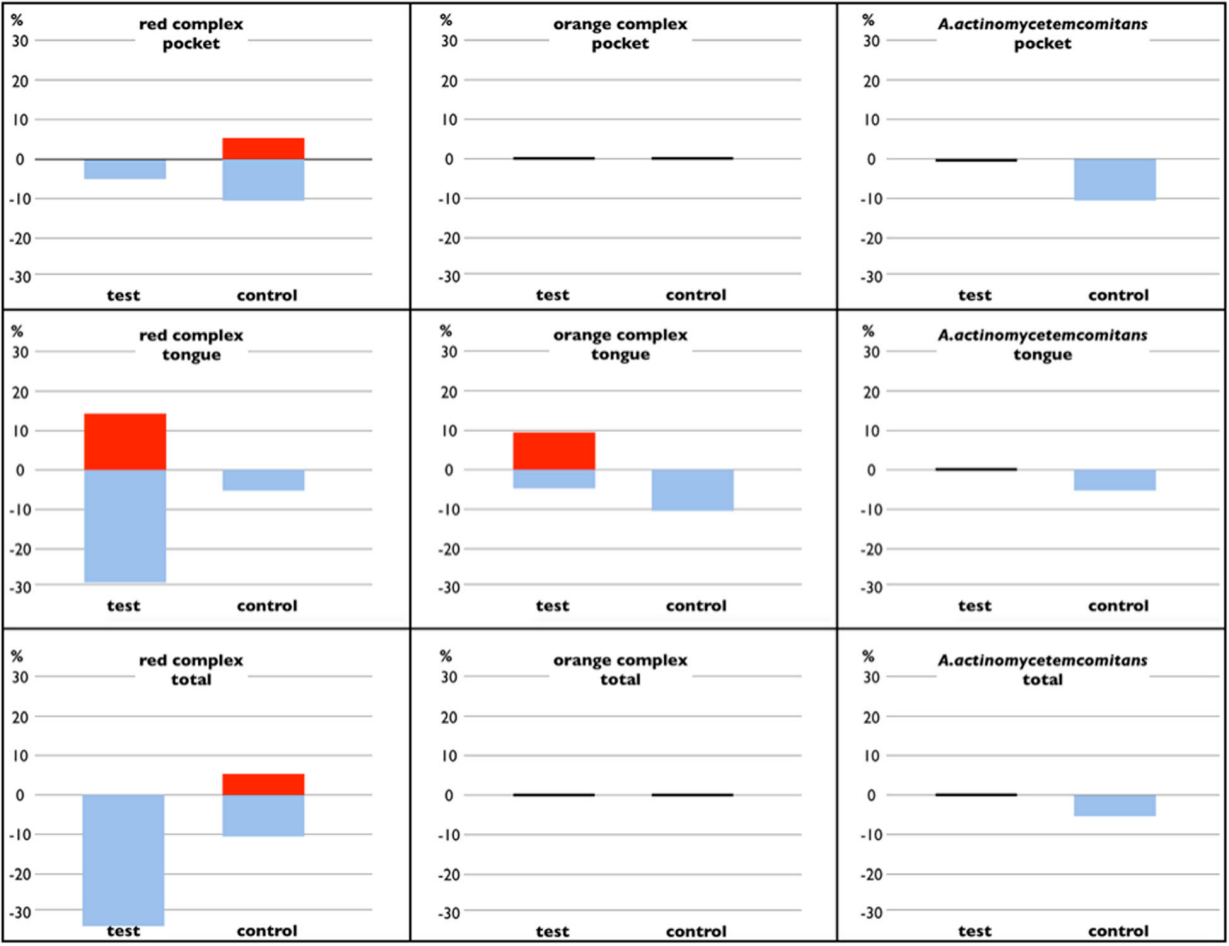
Changes of cases with the positive detection of red complex, orange complex bacteria and *A. actinomycetemcomitans* in different sites for the entire collective. The blue bars indicate a decreased percentage of cases with a positive detection, the red bars an increased percentage. No bars indicate no change

Changes for the presence/absence of the bacterial species from different sites were also analysed. Changes in the number of cases with a positive detection of red and orange complex bacteria and *A. actinomycetemcomitans* after six weeks are depicted in Fig. 1. None of these changes showed significant differences between the groups.

**Fig. 2:**
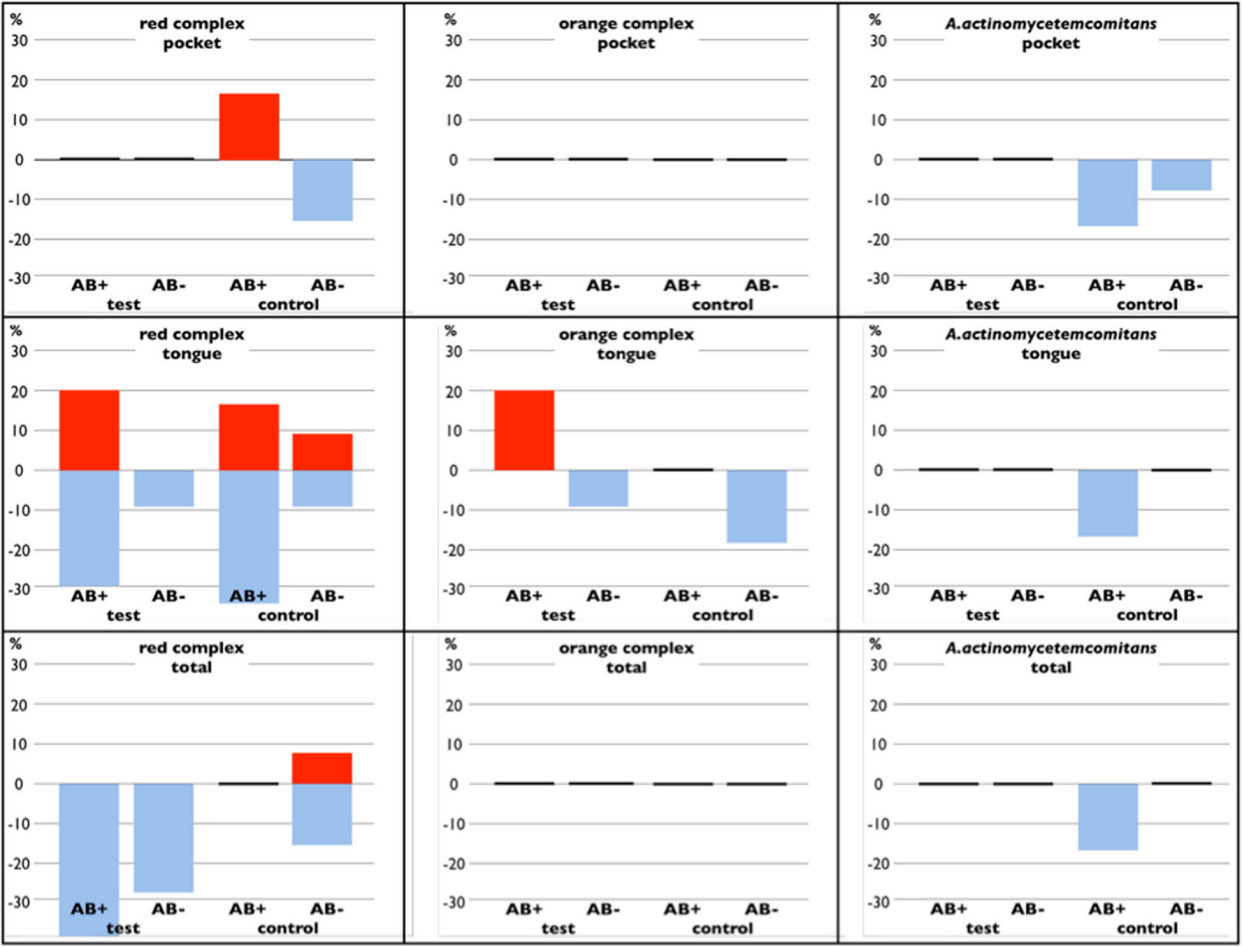
Changed numbers for the detection of red and orange complex bacteria and *A. actinomycetemcomitans* in different sites for the cases that have not been treated with antibiotics after intervention. The blue bars indicate a decreased number of detection, the red bars an increased number. Missing bars indicate no such cases

**Fig. 3:**
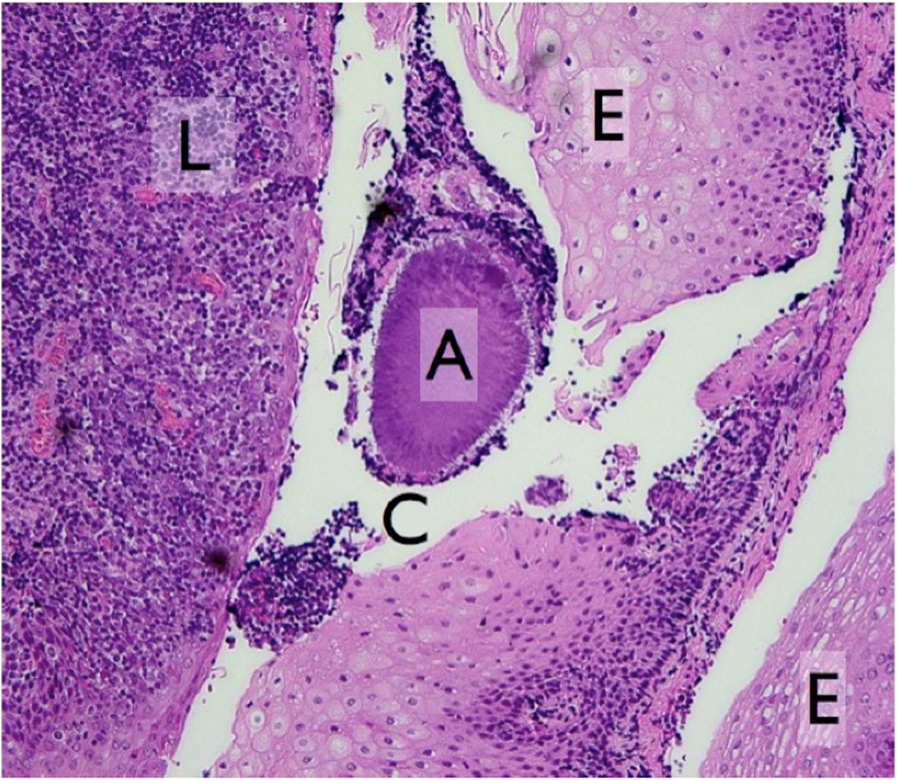
Histological section from a removed tonsil; C – crypt, L – lymphocytes, A – Actinomyces Druse, E – Epithel

A sub-group analysis of the bacterial changes of those cases, which were not treated with systemic antibiotics after the surgical intervention was also performed (Fig. 2). In accordance to the entire collective no significant differences were found regarding the presence of the individual bacterial species. Figure 3 shows a histological section from a removed tonsil.

## Discussion

Intraoral niches like tonsils have already been suspected to act as sources of reinfection for previously debrided periodontal sites [21]. In order to test this theory we assessed the taxa of oral key pathogens before and six weeks after tonsillectomy. However, as compared to a control group without tonsillectomy, no substantial differences could be found in terms of shift in bacterial flora in the present study. This was also valid for patients who had been treated with antibiotics during the observation time. Therefore, and with all limitations of the study in mind, we could not find our hypothesis confermed.

Numerous studies of a research group from Leeuwen are based on the fact that periodontopathogens can not only be detected in infected periodontal pockets, but also in other intraoral niches such as mucosa, tongue or palatine tonsils, and hence might have a crucial impact on periodontal therapy as stipulated by Quirynens’ full mouth disinfection theory [32]. The authors could demonstrate a more pronounced microbiological shift toward a beneficial flora two months after intervention, when not only scaling and root planing had been performed but also an addititional intensive mechano-chemical treatment of these oral niches outside the periodontium was applied [21]. Among these niches, the tonsils were supposed to play a key role.

Accordingly, the removal of tonsils – including respective biofilms - should show a beneficial effect on the flora of both, the teeth and the tongue. The present study assessed potential microbiological alterations after tonsillectomy. The results of this study, however, showed a more or less unchanged oral flora after tonsillectomy. Nevertheless, *Porphyromonas gingivalis* and *Tannerella forsythia* were reduced in the samples taken from the tongue. A possible explanation for this finding is the anatomical vicinity of tongue base and tonsils, which continuously touch during swallowing and speaking. A possible impact on the flora of the periodontal sulcus, which is more distant and maybe less accessible due to stable sulcus fluid flow might be less pronounced when considering limitations of investigation period and samples size. In addition, no periodontal therapy was performed.

Furthermore, some single species out of the eleven investigated species, *T. denticola, P. intermedia, C. rectus and E. corrodens,* were reduced significantly more in the test group. *T. denticola* is a periodontal pathogen belonging to the red complex. It is a Gram-negative and obligate anaerobic, motile spirochete. While these species are rarely found in a healthy periodontium, it is usually present in high numbers in sites affected by periodontitis. Likewise, obligatory anaerobe bacterial flora is present in tonsils with recurrent inflammation [22]. It is known that they can express surface proteins associated with adherence and degrading proteases [37, 38]. With regard to mobility and adherence, a decrease of *T. denticola* after tonsillectomy seems reasonable while transmission from tonsillar niches is impeded. *P. intermedia* belongs to the orange complex which is closely related to the red complex. It represents a gram-negative, black pigmenting obligate anaerobe periopathogen. Like *T. denticola*, *P. intermedia* can be found more often in infected periodontal pockets. Likewise, due to the expression of various adhesion proteins a transmission from harbouring niches like tonsils seem reasonable, and its absence render an association with tonsillectomy plausible [39].

*C. rectus* was described as a component of the orange complex as well. It is Gram negative and since it is a facultative anaerobic species a translocation by migration through the oxygen-saturated oral cavity from one niche to another is conceivable [3].

*E. corrodens*, a Gram negative facultative anaerobic bacillus and allocated to the green complex, plays a presumably important role as an early colonizer as well [3]. It is also meant to be associated with the development of periodontitis [40].

Our findings therefore partly contradict Quiryinen’s assumption, although several obvious limitations render a proper discussion necessary. The six weeks of observational period in the present study might be considered rather short for a shift in the bacterial composition. However, designing the present study we relied on the original investigation time by Quirynens et al. [21], where a significant impact of the extraoral niches was reported.

The present study was not limited to patients with periodontitis in order not to reduce the sample size of an already most special group of tonsillectomy patients. Unfortunately, there was a rather high number of ten dropouts, four from the test and six from the control group, for the second appointment. This is partly attributable to the fact, that the follow-up appointment was not linked to the medical treatment. Therefore, the follow-up appointment was an extra date without any personal benefit. In addition, for organizational reasons the second appointment had to take place in another Institution, that was previously unknown to most patients. Furthermore, as a standard of our ethical policy, patients were well aware that the participation in this study was strictly facultative and could be suspended at any time without any justification. Therefore, the number of participants with periodontal issues was rather low and, accordingly, the power of our findings may be considered as weak. As this study is the first of its kind, no reasonable power analysis could be performed beforehand. Nevertheless, it is important to highlight that though the result of the statistical tests show no significant difference, it cannot be concluded that there is definitely no influence of tonsillectomy on oral microbiota.

The design of the present study did not provide any therapeutic measures like mechanical destruction of the oral biofilm, which might have promoted a quicker bacterial shift after tonsillectomy and corroborate the FMD theory.

As a very important aspect, patient characteristics of the test and control group unfortunately differed in age and number of periodontal pocket depth > 4 mm. Since mostly children and adolescents suffer from recurrent tonsillitis or pharyngotonsillitis [27, 41], tonsillectomy is performed predominantly in younger patients. Although only adults were included in our study, it is in the nature of the regarding pathologies that the mean age of the control group turned out to be higher, since we only included patients with planned interventions. For the same reason the significant higher amount of periodontal pockets > 4 mm in the control group can be explained by this age disparity, as the prevalence of periodontitis increases with age [15, 42]. Furthermore, several microbiological studies showed that *A.actinomycetemcomitans* is associated with active and deep periodontal pockets [43, 44]. Thus, our results are still in line with these findings, as *A.actinomycetemcomitans* was found more often in the control group, where patients had more periodontal pockets exceeding 4 mm. Therefor it is questionable, whether the inclusion criteria of the control group should have matched better regarding the age of the patients. Likewise, using a perfectly age-matched but effectively untreated control group would have provided at least the bias of different physical and psychological stress. However, with the choice of an ambulant surgery of comparable invasiveness at in the same centre as the test group the confounding intergroup factors were kept as small as possible.

The information for the medical history, especially drugs and smoking habits, were self-declared by the patients. When interpreting the results of the present study it must be taken in consideration that this data might therefore be biased.

An important fact that has to be considered regarding the statistical analysis is that no distinct numerical values were available laboratory analysis. Due to the score-like semiquantitative analysis report of the bacterial test all calculations rely on either medians replacing the according range values of the test (Table 1) or on the dichotomous detection (or no detection) of the individual species. This shortcoming renders sophisticated statistical analysis like post-hoc power calculation or a regression analysisin order to test for confounders pointless. Anyhow, since the semi-quantitative DNA-strip test has been used in several previous studies it was shown to be a reliable test for bacterial analysis [36, 45]. Therefore, it constitutes a viable fundament for a basic analysis of how the oral flora might change after tonsillectomy as determined in this study.

The postoperative intake of antibiotics also turned out to be much higher than expected by the otorhinolaryngologic department. Postoperative infection rate after tonsillectomy has been reported to be about 26% in scientific literature [46]. However, it is a well-known problem that there are no mandatory guidelines for the diagnosis of a postoperative infection. Therefore, stringent indications for the administration of antibiotics in case of postoperative complaints are missing [47], which contributes to antibiotic over-therapy by general physicians. Among the administered antibiotics for the patients of the present study the most prescribed was Amoxicillin, an aminopenicilline effective on both Gram-positive and negative bacteria, which it is often administered to fight mixed infections like tonsillitis, pharyngitis and laryngitis in Switzerland. However, its effect on anaerobe bacteria like red complex species is weak. Therefore, its administration might have had a very limited consequence for our data. Each Cefuroxim and Clindamycin were administered only once in each group. Cefuroxim is prescribed for tonsillitis and pharyngitis. Clindamycin is effective against Gram positive aerobs and some Clindamycin-sensible anaerobes like in particular *Bacterioides* spp., what might have had an impact on our data. Noteworthy, at the second appointment bacterial taxa of patients with or without postoperative intake of antibiotics in the meantime showed no significant differences for any species when compared to patients without antibiotic intake. This finding corresponds with the results of a review on periodontitis treatment by Herrera et al., who showed that systemic antimicrobials should not be used without mechanical debridement [48] since intact biofilm is not subjected to substantial effects under pharmacologic attack. Also Fux and co-workers advises to employ additional measures in combination with antibiotic therapy when treating diseases caused by biofilms. The main problem is, that bacteria imbedded in the biofilm infrastructure are much more tolerant to antibiotics than planktonic bacterial species [49]. Several mechanisms have been found to be responsible for the strong resistance of bacteria embedded in biofilms [50–52]. Among these, the restricted diffusion of antibiotics through extracellular polymeric barrier, efflux pumps or the ability to alter metabolism are usually highlighted in literature [53]. However, this finding contrasts results from a clinical trial of Lopez et al. [54], which suggests that antibiotic administration has a similar beneficial effect on chronic periodontitis as conventional scaling and root planning.

## Conclusion

Based on the results of the present study and considering its various limitations, no indication for an immediate effect of sole tonsillectomy without any periodontal treatment on the bacterial flora of the tongue or periodontium could be found. More research on this topic is therefore required.

## Abbreviations

BoP: Bleeding on probing
FMD: Full mouth disinfection
PPD: Periodontal pocket depth
